# Building Social Networks for Health Promotion: Shout-out Health, New Jersey, 2011

**DOI:** 10.5888/pcd10.130018

**Published:** 2013-08-29

**Authors:** Pamela Rothpletz-Puglia, Veronica M. Jones, Deborah S. Storm, J. Scott Parrott, Kathy Ahearn O’Brien

**Affiliations:** Author Affiliations: Veronica M. Jones, Deborah S. Storm, J. Scott Parrott, Rutgers, The State University of New Jersey, Newark, New Jersey; Kathy Ahearn O’Brien, Hyacinth AIDS Foundation, New Brunswick, New Jersey. At the time of the study, Dr Rothpletz-Puglia was affiliated with the Francois-Xavier Bagnoud Center within Rutgers, The State University of New Jersey (formerly the University of Medicine and Dentistry of New Jersey).

## Abstract

**Background:**

Building social networks for health promotion in high-poverty areas may reduce health disparities. Community involvement provides a mechanism to reach at-risk people with culturally tailored health information. Shout-out Health was a feasibility project to provide opportunity and support for women at risk for or living with human immunodeficiency virus infection to carry out health promotion within their informal social networks.

**Community Context:**

The Shout-out Health project was designed by an academic–community agency team. During 3 months, health promotion topics were chosen, developed, and delivered to community members within informal social networks by participants living in Paterson and Jersey City, New Jersey.

**Methods:**

We recruited women from our community agency partner’s clients; 57 women participated in in-person or online meetings facilitated by our team. The participants identified and developed the health topics, and we discussed each topic and checked it for message accuracy before the participants provided health promotion within their informal social networks. The primary outcome for evaluating feasibility included the women’s feedback about their experiences and the number of times they provided health promotion in the community. Other data collection included participant questionnaires and community-recipient evaluations.

**Outcome:**

More than half of the participants reported substantial life challenges, such as unemployment and housing problems, yet with technical support and a modest stipend, women in both groups successfully provided health promotion to 5,861 people within their informal social networks.

**Interpretation:**

Shout-out Health was feasible and has implications for building social networks to disseminate health information and reduce health disparities in communities.

## Background

Disseminating preventive health information through informal personal networks can reach at-risk community members and may create social networks that reinforce healthy behaviors in high poverty areas (1,2). When community members define the relevant health-related topics and take part in constructing and disseminating materials, the results are effective messaging and “community relevant” efforts to reduce health disparities (3,4). Community choice enhances the local relevance and contributes to an ecologic approach to health promotion (5).

Community-participatory and peer-led efforts for reducing health disparities benefit the community (6–9). In addition, community-participatory methods of disseminating preventive health behavior information may benefit community change agents. Opportunities for community members to learn about health and to master skills, such as providing health promotion, promotes self-efficacy (10,11). Self-efficacy can be seen as integral for health self-care with consequent health improvement or prevention. For example, if a woman living with human immunodeficiency virus (HIV) infection improves her self-care to include medication adherence, she will improve her immune status and health, and can decrease HIV transmission (1). Fostering skills to promote community health behaviors may empower change agents to improve their own health behaviors and increase the social impetus for them to do so because of increased pressure on change agents to “practice what they preach.” Therefore, a program that creates opportunity for community members to help others should prove mutually beneficial to participants and their communities. We developed the Shout-out Health program to engage women in community-designed relevant health promotion outreach.

## Community Context

In 2011, our academic–community agency partnership between Rutgers University (formerly the University of Medicine and Dentistry of New Jersey) and Hyacinth AIDS Foundation tested the feasibility of women at risk for or living with HIV infection to provide health promotion within their informal social networks through the Shout-out Health program. Because HIV/AIDS trends in the United States depict concentrated “hot spots” — geographic areas where HIV/AIDS is the leading cause of death among women of color, with a significantly higher proportion of HIV infections occurring in areas with high poverty rates (2,12) — we tested the feasibility of Shout-out Health in Paterson and Jersey City, New Jersey. We chose these 2 because more than a third of residents in both cities are racial/ethnic minorities; 16% of residents in Jersey City and 27% of residents of Paterson earn an annual income below the federal poverty guidelines (13). Our academic community partner team consisted of members from a state university with experience in HIV care and treatment and a community-based agency that provides HIV prevention and treatment services within the targeted communities.

The objective of the Shout-out Health intervention was to offer opportunity and support for high-risk community members to identify, create, and provide health promotion messaging within their informal personal networks. Community engagement objectives for the project included developing the academic–community agency partnership to facilitate Shout-out Health and developing a community-driven health promotion approach based on the local realities of women at risk for or living with HIV infection.

## Methods

The academic–community partner team developed the Shout-out Health program procedures. The François-Xavier Bagnoud Center Community Advisory Board reviewed the program, and the Rutgers University (formerly the University of Medicine and Dentistry of New Jersey) Institutional Review Board approved the program. Our community agency team members provided advice about the 2 cities to target for the Shout-out Health intervention, and the inclusion criteria included English-speaking, adult women. We recruited women from the community agency client base. The community agency serves women at risk for or living with HIV infection. The community agency definition for women at risk for HIV infection includes self-reported active engagement in behavior that places them at direct risk of HIV infection, such as needle sharing and unsafe sex. Community outreach workers recruited women and invited them to a meeting with the program team to learn more about participating in Shout-out Health.

Sixty-five women from the 2 cities consented to take part in Shout-out Health. Fifty-seven (87%) of these women completed both pretest and posttest questionnaires. Of the 8 participants who chose to stop participating, 5 were from the in-person meeting group and 3 were from the online meeting group.

Of the 57 women who completed Shout-out Health, 38 chose to take part in the in-person meeting group and 19 chose to meet in the online group. We offered the choice of the online or in-person meeting approach because we had already feasibility-tested Shout-out Health in Newark, New Jersey, with 14 women randomized to an online or in-person meeting group and there were no differences in participation by meeting approach. Therefore, for the participant’s convenience, they could choose the meeting format of preference but could not move back and forth between the 2 approaches.

Most participants were racial or ethnic minorities, and because of the nature of discussion about health self-care, they self-identified as HIV-infected. We collected data about psychosocial factors, indicating that the participants experienced substantial life challenges: 66.1% had at least a high school diploma; 76.8% were unemployed; 60% had problems with housing sometimes, most, or all the time; and 22.4% reported that they experienced a low mood or depression most or all the time.

We based the meeting process on empowerment education principles (14–16). Steps in the process were as follows:

1. Identification of health issues each participant felt were important to women in their community. Women’s opinions of important health issues were not prioritized, though the groups discussed each topic when the participant explained why the health topic was important.
2. Discussion and development of the health promotion topic, including materials for distribution. Participant knowledge and information for dissemination were evaluated for accuracy and supported by the academic–community agency team.
3. Development and discussion about how the participant would provide health promotion in the community. This included role-playing and discussion about the participants’ personal action plans, strategies, and tracking plans for health promotion encounters in the community. We defined an encounter as a participant’s interaction with a community member to promote the health topic. Participants were not required to conduct any particular number of health promotion encounters.
4. Participants provided health promotion within their informal social networks and communities for 1 and one-half months.
5. Participants met once after the health promotion in the community concluded to discuss and report on the community health promotion experiences.

We designed this 5-step intervention process (Table 1), with 2 groups: a traditional, in-person meeting group and an online meeting group. The in-person meeting group convened 4 times for 2 hours, over a 3-month period. During the same period, women in the online group worked asynchronously except for 2 conference calls. All participants provided health promotion outreach on their selected topics to women in their informal social networks over a 5- to 6-week period. Women received gift cards as reimbursement for their time, travel, and child care expenses: $50 at step 2 and $100 at each of step 3 and step 5.

**Table 1:** The Shout-out Health Promotion Project Process and Procedures, New Jersey, 2011

Step	In-Person Meeting Group	Online Meeting Group	Measures
**Baseline**	Recruitment and consent meeting	Recruitment and consent meeting	Questionnaire
**Step 1: Week 1**	Meeting 1: discuss self-care of health issues	Asynchronous online discussion blog and live teleconference call to discuss self-care of health issues	NA
**Step 2: Weeks 2–6**	Meetings 2 and 3: select and develop self-care of health topic	Asynchronous online discussion blog to select and develop self-care of health topic	
**Step 3: Weeks 5–6**	Meeting 3: finalize materials and plan community outreach	Asynchronous online discussion blog to finalize materials and plan community outreach	
**Step 4: Weeks 7–12**	Provide promotion of health self-care in the community	Provide promotion of health self-care in the community	Community survey
**Step 5: Week 12**	Meeting 4: discussion on community encounters	Asynchronous online discussion blog and live teleconference to discuss community encounters	Questionnaire

Abbreviation: NA, Not applicable.

Health promotion encounters were self-reported in activity logs and by accounting for print materials that were distributed. Encounter quantity was the primary productivity outcome measure, though the qualitative analysis of the participant’s discussions about health promotion encounters provided information about the context, time, and type of encounters. Quantitative preprogram and postprogram questionnaires included an empowerment score from an empowerment scale developed by Rogers et al (17), and participants reported preventive health behaviors at baseline and step 5. All meetings were audio-recorded for qualitative analysis. The 21-item survey has 9 questions about various self-care behaviors such as Papanicolaou screening and dental care; 5 questions related to influences on self-care behavior such as food security, substance abuse, and housing; 2 questions about health care provider relationships; 1 question about caring for children or families (a broad gender role and societal expectation); and 4 questions about demographics such as employment and level of education.

The second part of the pre-post questionnaire consists of the empowerment scale for assessing personal and collective empowerment outcomes (17). Rogers et al developed and validated the Empowerment Scale in a mental health population; we selected the tool because it consists of 4 domains that captured outcomes of interest for this program (self-esteem/self-efficacy; power/powerlessness; community activism and autonomy; and optimism or control over the future), and we did not find an alternative tool for the general community in the literature. We did not revalidate the tool with our population, nor did we calculate scores for participants who skipped an item in the empowerment scale. We used descriptive statistics, *t* tests, and nonparametric tests to examine overall changes and group differences in the pretest and posttest Empowerment Scale score and for self-care behavior changes. Two team members sorted, coded, and analyzed the qualitative data for themes.

Participants also requested that the recipients of their health promotion evaluate the encounter, and we provided an information sheet and a telephone number to call us to complete the evaluation by telephone.

## Outcome

The health promotion provided by the 57 participants reached 5,861 people in the 2 cities. The number of outreach encounters ranged from 1 to 450, with a mean of 103 outreach encounters per participant. We saw no significant differences in the productivity by group meeting format.

Participants identified 14 different topics as important for health promotion within their informal social networks; the most frequently chosen health topic was HIV and sexually transmitted disease (STD) prevention (n = 12), followed by depression prevention and treatment (n = 11), and substance abuse treatment (n = 9). Other topics chosen by 5 or fewer participants were stress management, discussions about the choices we make about health, smoking cessation, breast cancer screening and awareness, a church member health needs assessment, nutrition and food acquisition, family violence prevention, senior health, sleep hygiene, services for women who were recently released from prison or women living with HIV infection, and communicating with health care professionals. Nine participants in 1 city worked in groups of 2 or 3 to deliver their health promotion in the community, and the remaining 48 developed an individual approach to community health promotion for their topic.

Most of the participants targeted specific locations for their health promotion efforts, such as streets with active drug use or prostitution. One participant characterized the area she went to provide health promotion to friends about substance abuse treatment as the “devil’s mouth” — a street formally known to the participant. Likewise, many other participants targeted community members with known problems. Furthermore, because of their relationships within the community they also provided information to the targeted women’s relatives to attain family support for an issue.

Other action plans included striking up conversations at the beauty parlor, community events, family gatherings, and other community locations. Much of this health promotion deliberately capitalized on existing informal social networks and community connections — for example, an older Hispanic woman developed a plan to help her Hispanic peers learn to communicate more effectively with their children or grandchildren about HIV and STD prevention.

The individual contact for health promotion was widely variable and innovative. One participant provided information about how to conserve food dollars to people waiting in line at soup kitchens and while people dined at soup kitchens. Another participant gathered her group for a discussion about treatment of depression by providing text messaging outreach. One participant provided information and facilitated discussion about family violence prevention during a retail store staff meeting; this led to other retail store owners requesting that this participant provide information at their staff meetings. Four participants distributed information at health fairs; 1 of these participants convened her own health fair and arranged for free US Public Health Service health information materials to be sent to her senior center. She drew women to the event by offering manicures, and after the health fair she set up a schedule to remind herself monthly to complete the online order forms to have health information sent to her senior center regularly. This participant arose as a natural leader in the group, and she facilitated participant involvement and community health promotion even after she completed the Shout-out Health program.

The preprogram and postprogram questionnaires revealed no significant change in empowerment score from baseline to postintervention overall, by group meeting format, or by city (Table 2). However, the participants we qualitatively categorized within self-determination theory constructs as autonomously motivated had significantly more encounters (mean [SD], 127 [95]) than participants in the controlled motivation group (51 [30]; *P* < .001). Self-determination theory holds that autonomous motivation for an activity arises from eagerness because of a sense of importance, whereas controlled motivation for an activity is due to the compensation or expectations of others (18). In addition, 4 themes coded by 2 team members with 100% agreement during content analysis of the meeting transcripts and online discussion blogs arose: community unity and activism, self-efficacy and confidence, perceived power, and optimism. Examples of the passages exemplifying the 2 most prominent themes of community unity and self-efficacy are in the Box.

**Table 2:** Changes in Questionnaire Empowerment Scale Scores From Pretest To Posttest Overall and Differences by City and Group, New Jersey, 2011

Characteristic	Number^a^	Empowerment Score^b^			*T* c	*P* Value
		Preintervention, Mean (SD)	Postintervention, Mean (SD)	Change From Preintervention to Postintervention, Mean (SD)		
Overall change	38	2.0 (0.29)	2.0 (0.33)	0.06 (0.25)	1.53	.33
Paterson	19	2.0 (0.27)	2.0 (0.39)	0.01 (0.27)	0.26	.79
Jersey City	19	2.0 (0.31)	1.9 (0.27)	0.11 (0.28)	1.73	.10
In-person meeting group	29	2.0 (0.27)	2.0 (0.32)	0.05 (0.26)	0.99	.33
Online meeting group	9	2.0 (0.36)	1.9 (0.40)	0.11 (0.23)	1.44	.18
Autonomous motivation^d^	28	2.0 (0.27)	1.9 (0.29)	0.10 (0.24)	2.22	.03
Controlled motivation^d^	10	2.0 (0.36)	2.1 (0.44)	−0.04 (0.26)	−0.52	.61

a Number of participants who completed each item within the Empowerment Scale.

b Possible scores ranged from 1 to 4.

c Paired *t* test.

d Qualitatively determined by the study facilitators within self-determination theory constructs.

Box. Participant Quotations Illustrating the 2 Most Prominent Empowerment Themes, Shout-out Health, New Jersey, 2011 Community Unity and Activism

“I felt — I felt great about it. It was okay because it was some stuff in here like I really didn't know about. And then to pass it on to somebody else that probably didn't know about it, I felt good about it.”

“I'm so grateful for everybody, because I wish if I would’ve known the way I’m teaching my kids, my children in the young community about this disease, probably I wouldn't be HIV positive if I would’ve knew this stuff, what I know now.”

“There were about 40 people there and we spoke about nutrition and then everybody put their piece in. It was a wonderful meeting. But after that, the word spread. I was invited to a women’s group.”

“We all came together to make a difference in the community.”

“The project was real women taking action and helping others just like themselves.”

“For some women health and wellness takes third or fourth place when faced with more pressing social issues such as poverty, unemployment, etc. This community campaign helped encourage women to take better care of themselves.”

“Knowledge is power and by sharing ideas we become more informed.”

Self-efficacy and Confidence

“I like that I created my own ideas and used them to help parents and teens become more aware.”

“The experience of brainstorming a message of our own choice and the ability to share that message with others was amazing.”

“Participating in the group had helped me to stay focused and committed on my own journey to health and fitness.”

“I found out information I did not know before and was able to relate to people. Before this, I thought I was on my own.”

“I like the fact that I got to help out people in need and got to talk to people and make new friends that I didn’t have before. It also helped boost my confidence to approach people and voice my information in a professional manner.”

“I enjoyed myself and had a lot of fun. This gave me confidence I did not have before and also it was an opportunity of a lifetime to do something for the community.”

We observed no significant changes in participant’s self-care of health behaviors preprogram to postprogram. However, themes related to participants’ increased knowledge and awareness about preventive health arose in the content analysis of meeting transcripts and online written dialogue. We coded 27 passages with 100% agreement as indicative of increased self-care knowledge and awareness. Example passages follow:

“I liked that I was able to participate in helping women with their health and I also learned valuable info for myself.”

“By helping women in the community I am motivated to help myself with the information that was discussed and provided in the folder that was handed out in the beginning of the research. I was given an opportunity to read the information and educate myself on women’s health issues. Some of which I knew about, others I did not know more about.”

Seventy-four community members who received health promotion information from participants called the study team to evaluate the encounter. Close to half of these evaluation respondents (n = 31) were friends of the participant, 8 respondents were family members, 8 were classmates, and 8 were other. The remaining respondents were strangers, neighbors, or church members. These evaluations indicated that the recipients of the health promotion found the information to be helpful, and the evaluations provided us with preliminary information about the participant’s social networks.

## Interpretation

The primary objective of Shout-out Health was to offer opportunity and support for community members to develop and provide health promotion information within their informal social networks in 2 cities with the goal of reducing health disparities. Within 12 weeks, 57 women at risk for or living with HIV infection developed diverse health promotion messages and disseminated them to 5,861 people in their communities, demonstrating the feasibility and effectiveness of the Shout-Out Health program approach. The sheer scope of this health promotion, and women’s abilities to find community members with particular health needs, is noteworthy and has implications for interventions that build social network opportunities for healthy behavior promotion by at-risk community members within hot spot areas.

Other objectives related to community engagement included establishing a strong academic–community agency partnership to facilitate Shout-out Health and a community-driven approach to health promotion. This project’s success is in part attributable to participation from both the academic and community partners. The academic partner procured funding for the project and provided expertise on program design and evaluation. The community agency partner provided the knowledge and awareness of the targeted communities and enabled an approach based on local realities. Both partners participated in the facilitation and management of Shout-out Health and worked together with mutual respect. The final community engagement objective was to create a community-driven health promotion opportunity for women to take part in. Participants identified 14 health issues that they considered important for their community. Some of the materials developed for Shout-out Health and basic strategies for community-participatory health promotion can be found at http://onlineworkgroup.ning.com/. Participants also developed numerous strategies for promoting their health topic within their neighborhoods and social networks. Many of these approaches are innovative and resulted in sustainable health information dissemination networks as exemplified by the aforementioned description of the woman who set up a calendar and facilitated free US Public Health Service health information to be routinely sent to her senior center for health fairs. Our strong academic–community partnership is related to the success of this community-driven approach to health promotion, but the success is also attributable to the participants’ visions for improving health and reducing health disparities in their communities.

We recruited participants from our community agency partner centers where they serve women at risk for or living with HIV infection. With this targeted recruitment, women participants reported many of the problems experienced by others in their communities — unemployment, housing problems, chronic illness, and depression. Therefore, the personal, informal social networks of these participants included at-risk and difficult-to-reach community members. Participants reported they were close to their target populations, and the 74 evaluations completed by the recipients of the health promotion support this. This is consistent with findings from other community-participatory health promotion efforts where participants deliberately capitalized on existing informal social networks and community connections (7,19). These close relationships may foster trust and credibility (20) and health promotion messages that are relatable because participants understood their social network’s needs and norms. Not only did these participants provide widespread health promotion in their informal social networks but also the recipients of the health messaging were at-risk community members in need of preventive health information.

Shout-out Health provides preliminary evidence for assessing the personal gain of participants in community engagement projects. Findings from the Shout-out Health community project show that given support, discussion, training, and information, at-risk community members can successfully provide health promotion to other high-risk community members and may personally benefit by helping others in their community. Future programs like Shout-out Health should include the evaluation of participants’ personal gains, community-member health outcomes, and the long-term and sustainable effects of building social networks for health.
